# Emergency Medicine: On the Frontlines of Medical Education Transformation

**DOI:** 10.5811/westjem.2015.8.28393

**Published:** 2015-10-22

**Authors:** Eric S. Holmboe

**Affiliations:** Accreditation Council for Graduate Medical Education, Chicago, Illinois

## Abstract

Emergency medicine (EM) has always been on the frontlines of healthcare in the United States. I experienced this reality first hand as a young general medical officer assigned to an emergency department (ED) in a small naval hospital in the 1980s. For decades the ED has been the only site where patients could not be legally denied care. Despite increased insurance coverage for millions of Americans as a result of the Affordable Care Act, ED directors report an increase in patient volumes in a recent survey.^1^ EDs care for patients from across the socioeconomic spectrum suffering from a wide range of clinical conditions. As a result, the ED is still one of few components of the American healthcare system where social justice is enacted on a regular basis. Constant turbulence in the healthcare system, major changes in healthcare delivery, technological advances and shifting demographic trends necessitate that EM constantly adapt and evolve as a discipline in this complex environment.

In this context the emergency medicine (EM) residency community has embraced the challenge to transform training for the 21^st^ century. It is probably no accident EM was one of just five specialties to implement and report on Milestones during the 2013–2014 academic year. And it is also no surprise EM will publish the first validity study on Milestones.^2^ How did EM get to this point? In this commentary, I hope to accomplish three objectives. First, I will briefly review the history of the next accreditation system (NAS), competencies and Milestones. Second, to provide an “outsider’s view” of why implementation of competency-based training, and specifically the Milestones, appears to be off to a healthy start in the EM community. Finally, I will offer some thoughts on further steps necessary to realize the full potential of competency-based medical education in U.S. residency training because much work remains to be done. We are moving from a “static/stable” view of educational programs to one that is dynamic and constantly evolving. The Milestones, along with the Clinical Learning Environment Review, are regulatory representations of this shift; both are designed to be formative, continuous quality improvement components of the NAS.

## A Brief History of Competencies, Milestones and the NAS

The NAS was part of the educational community’s response to improve graduate medical education (GME).^3^ The NAS is designed to help achieve the original vision of the Outcomes Project that was officially launched in 2001, based on the six general competencies formally approved by the Accreditation Council for GME (ACGME) and the American Board of Medical Specialties (ABMS) in 1999.^4^ However, programs struggled to implement an outcomes-based approach and operationalize the competencies, new concepts to many medical educators, into meaningful changes in curriculum and assessment.

One reason for this struggle is the lack of shared mental models, or frame of reference, regarding the competencies among programs and clinical faculty. There were several reasons for this struggle. First, the ACGME/ABMS general competencies were defined in conceptual terms that were often hard to translate into practice. Second, some of the competencies, especially practice-based learning and improvement and systems-based practice, were new concepts altogether. Third, work-based assessment methods were either unavailable or not well aligned with the purpose and goals of the competencies.

The Milestones were developed collaboratively by each specialty to create the core blueprint, or roadmap, of the discipline in narrative, developmental language. In other words, they have helped to describe the competencies in more understandable language. The Milestones serve as a framework to inform and guide curriculum, choice of assessment methods and instruments, and assessment judgments by the clinical competency committee.^5^ Milestones also begin to move us away from an over-reliance on the quantification of competence, traditionally represented by numeric rating scales, toward a more qualitative, descriptive approach.

There are two important caveats. First, milestones do not define the totality of any discipline, including EM. Milestones are key elements of a larger “whole” of clinical competence. Second, substantial professional judgment, on the part of the faculty, is critical in the overall assessment of readiness for clinical practice. Informed judgment, based on multiple assessments through various forms of observation, is a cornerstone of a competency-based system.^6^ Using Milestones to guide and perform systematic measurement holds promise to enhance our ability to assure the public of the effectiveness of GME to prepare physicians for practice.

## Milestones and EM Training

EM was one of the early adopters in the NAS, being just one of five specialties to report both mid-year and end-year Milestones data in the 2013–14 academic year. Several aspects of the EM approach to developing and implementing the Milestones are noteworthy. First, the Milestones are grounded in the “Model of the Clinical Practice of EM” (EM Model). The EM Model consists of three core components: 1) individual conditions; 2) physician tasks; and 3) acuity levels. The knowledge, skills and attitudes comprising the current EM Model were informed by a national survey of 9,740 physicians in 2007 regarding EM practice.^7^ Second, the American Board of EM (ABEM) engaged over 60% of EM residencies as part of a national validation study. Participating program directors essentially took the EM Milestones for a “test drive” and provided the EM Milestones working group with feedback on the Milestone placements and descriptions. In fact, of the final 227 Milestones included in the 23 subcompetencies, 46 Milestones were reassigned different performance levels based on the program feedback.^2^,^7^

The EM approach to Milestone development appears to have had an important impact. The initial validity study of the EM Milestones reported in year one shows very promising results. Factor analysis of the national Milestones data revealed a three-factor structure concordant with the three component EM Model of practice.^2^ In addition, reliability coefficients for the Milestones were robust.^2^ My hypothesis for these early positive findings brings me back to one of the initial purposes of the Milestones: to create shared mental models of the general competencies not only within EM residencies, but equally important between EM residencies across the country.^7^ EM will likely stand as an exemplar for other specialties in how to build national standards for judging EM residents. In essence, the EM developmental approach is akin to a nationally-based performance dimension training (PDT) exercise. PDT is an established approach to helping to improve performance evaluations by getting all evaluators “on the same page.”^6^,^8^ By incorporating empiric evidence into EM certification design along with robust involvement of educators and program directors, the EM educational community has already likely made substantial progress in creating shared mental models of EM training and assessment.

## Where Next?

The NAS is built on a foundational principle of continuous quality improvement. In the United States and most of the world, education and healthcare systems are experiencing significant change and disruption. We must continue to move away from a “static/stable” view of education and clinical care to one that is dynamic and constantly evolving. There are several implications for GME. First, changes in educational programs must become better integrated with the changes occurring in healthcare delivery and systems. Care of patients and populations is a dynamic, integrated process. As the frontline specialty of the healthcare system experiencing this disruption, EM is well positioned to lead and inform educational redesign. Second, work-based assessments will continue to grow in importance and prominence. One example of a useful technique in EM is end-of-shift encounter cards.^9^ When used properly, encounter cards can enhance the quality of assessment and feedback. EM also leads the way in the development of chart stimulated recall (CSR), a validated method to assess clinical reasoning of actual patient care.^9^ While not currently in widespread use, CSR and other performance-based methods (e.g. clinical indicators and patient experience) represent the next frontier in work-based assessment for EM training.

Third, the current Milestones are truly version 1.0; as in all continuous quality improvement processes some amount of change and revision will be needed. The processes used by the EM community to create version 1.0 will be invaluable to the larger educational community and capturing the detail behind these processes will be important. Finally, we want to ensure the Milestones do not create overly reductionistic assessments and curricula. Residency education will be most effective when the output is a whole physician who effectively integrates all competencies, however defined, into his or her practice. For example the EM community has developed entrustable professional activities (EPAs) to further help operationalize the competencies and milestones.^10^,^11^ EPAs hold promise to help enhance curriculum and assessment, using milestones as “building blocks” for each EPA. Our collective goal is to produce physicians who can successfully enter unsupervised practice and continue their trajectory toward expertise and mastery. Competencies, Milestones and in the near future EPAs can serve as meaningful frameworks to help produce a talented, whole physician.

In conclusion, here is a request to the EM community: The ACGME, the ABEM and the dozens of talented EM faculty who volunteered their time, expertise, and wisdom to advance the Milestones, need and welcome constructive feedback to continually improve the NAS and Milestones. Milestones are tools to facilitate and promote innovation and continuous improvement in GME in the Unites States, but they are not yet fully realized and will require changes and adjustments. We are entering a period of transformation that requires collectivism among all the key stakeholders and that can feel, like any change, uncomfortable. Only by working together through dialogue and across organizations can the full potential of outcomes-based medical education be realized. The EM educational community has clearly taken up this charge.
